# Editorial: Advancing animal reproduction: Artificial Intelligence, precision technologies and reproductive biotechnologies

**DOI:** 10.3389/fvets.2026.1790388

**Published:** 2026-02-10

**Authors:** Charley-Lea Pollard, Nesrein M. Hashem, Sadanand D. Sontakke, Pierre Comizzoli

**Affiliations:** 1 Sydney School of Veterinary Science, Faculty of Science, The University of Sydney, Camden, NSW, Australia; 2 Nanoencapsulation and Biotechnology Lab, Animal and Fish Production Department, Faculty of Agriculture (El-Shatby), Alexandria University, Alexandria, Egypt; 3 Council of Scientific and Industrial Research-National Environmental Engineering Research Institute (CSIR-NEERI), Nagpur, India; 4 Office of the Undersecretary for Science and Research, Smithsonian's National Zoo and Conservation Biology Institute, Washington, DC, United States

**Keywords:** artificial intelligence, conservation, machine learning, precision technologies, reproduction, reproductive biotechnology

## 1 Introduction

Reproductive biotechnologies have revolutionized animal breeding programs over the last 50 years, continuing to be the driving force behind improvements in reproductive performance. The use of nanotechnology, precision technologies and artificial intelligence (AI) are still in their infancy in animal reproduction, but their application to breeding programs are steadily increasing as the world finds new ways to promote sustainability, enhance conservation, and ensure food security for decades to come, particularly under climate change challenges.

This Research Topic presents recent research conducted across the various types of technologies available in animal reproduction, including established and emerging livestock industries as well as conservation breeding programs. Seven papers included in this Research Topic explore the use of various technologies, from reproductive biotechnologies through precision technologies and AI, showcasing the diversity of research across species.

## 2 Reproductive biotechnologies

The bulk of research programs, particularly for new and emerging activities, heavily focus on improvements to reproductive biotechnologies, adapting methods already in use in established industries. However, there is now a shift toward the use of multiple types of advanced approaches on improvements of animal reproduction.

Esteve et al. examined the impact of seminal plasma in sperm quality following freeze-thawing in Murciano-Granadina goats, in response to the growing need to maintain endangered breeds while maximizing transport distances and time. The study showed seminal plasma is detrimental to sperm quality at the refrigeration step, while a significant reduction in the survival of sperm cryopreserved in the presence of seminal plasma compared with sperm cryopreserved without seminal plasma was also evident.

On the other hand, Rios et al. examined the impact of oocyte *in-vitro* maturation media and vitrification parameters on the quality of bovine oocytes following vitrification. Oocytes matured in serum-free media exhibited higher post-thaw survival through maintenance of membrane integrity irrespective of the vitrification device used, while oocytes matured in synthetic media and vitrified with a surface device exhibited higher post-thaw survival, highlighting the potential combination of technologies on reproductive outcomes.

## 3 Nanotechnology

Nanotechnology offers novel but versatile functions in animal reproduction, from finding new ways to deliver therapies and control reproductive function through its use in the development of biosensors. Shehabeldin et al. investigated the use of chitosan nanoparticles as a nano-drug delivery method for pregnant mare serum gonadotrophin (PMSG) during estrus synchronization protocols in Ossimi sheep. Pregnancy, lambing and overall fecundity rates were greater when hormones were delivered through nanoparticles, resulting in more efficient uptake through cell pores, membranes and greater binding affinity to follicle-stimulating hormone receptors. This technology is adaptable to other livestock oestrus synchronization protocols and previously demonstrated in goats (1).

## 4 “Omics” technologies

Genomic technologies are a key attribute of species management, particularly in conservation breeding populations exhibiting a greater risk of inbreeding and reduced population fitness. Genomic technologies aim to prevent the extinction of a species by maintaining genetic diversity within the population for future breeding populations. Chen et al. used next generation sequencing to determine kinship structure and relatedness in a captive lion population in China. The work showed genetic markers were effective at identifying distinct genetic subgroups within the captive population, proposing a suitable breeding plan to further reduce the risk of inbreeding and promote evolutionary potential. The findings of this work can be applied across other breeding programs to enhance conservation outcomes.

## 5 Systems biology and data driven techniques

A systems biology approach may help to integrate information generated through “omics” technologies with data driven analysis and predictions. Amelkina and Comizzoli propose a systems biology approach to transcriptomic analysis in domestic and wildlife species, highlighting publicly available data repositories relevant to advancing reproductive science and conservation programs. Difficulties with availability of public data with particular reference to a lack of expression atlases and digital biobanks for model species were identified, concluding that a greater number of reproductive expression databases and species-specific biobanks are required to strengthen evolutionary and conservation breeding programs globally.

## 6 Precision technologies and artificial intelligence

Precision technologies and artificial intelligence are often combined and widely implemented in horticulture and crop industries worldwide, only recently being explored in the animal reproduction industries. Precision technologies such as biosensors for monitoring reproductive physiology are steadily increasing while artificial intelligence for monitoring behavioral indicators of reproduction are more likely to be adopted earlier due to their wider and less invasive application.

Mansour et al. explored the use of AI-based video tools to monitor behavioral patterns of dromedary camels in real time during pregnancy, through parturition and beyond. Video based tools captured alterations in daily activity patterns in periparturient camels compared with pregnant camels, exhibited by identification of reduced eating, drinking, sitting and sleeping patterns, which gradually returned to normal postpartum. The technology allowed continuous monitoring of individually identified animals with the potential to scale the technology to larger herd sizes and greater complexity housing layouts.

Compared with standalone AI technologies, precision technologies and AI can also be applied to pre-existing reproductive biotechnologies. Kang et al. reviewed the current technologies associated with laparoscopic artificial insemination in sheep, including precision estrus detection, genomic selection, robotic-assisted surgery, semen optimization and wearable biosensors to measure physiological parameters and improve reproductive management. While the benefits of precision technologies and artificial technologies are numerous and well-documented, the cost of the technology and a lack of data standardization methods remain barriers in its adoption in the industry.

## 7 Final remarks

This Research Topic highlights current research in the animal reproduction industries, from the development of reproductive biotechnologies applicable to improving reproductive performance of a single species through to the application of newly emerging precision technologies, data driven methods and AI capable of being implemented across entire areas of activity. While the shift from reproductive biotechnologies is becoming increasingly evident, advancements in reproductive biotechnologies are still considered critical in all species. This Research Topic emphasizes this shift, with the expectation that future research will combine multiple technologies to further enhance reproductive performance necessary to promote sustainability, enhance conservation, and ensure food security.
